# Supplementary material for: Data-driven analyses of behavioral strategies to eliminate cysticercosis in sub-Saharan Africa
Source: PLoS Negl Trop Dis. 2021 Mar 23;15(3):e0009234. doi: 10.1371/journal.pntd.0009234 (PMC8018642; doi:10.1371/journal.pntd.0009234)
Supplement: S1 Equations — (DOCX) [file pntd.0009234.s001.docx]

**S1 Equations.** Transmission Model Equations.

Pigs

$$\frac{dS_{P}}{dt}=b_{P}N_{P}-\varepsilon S_{P}-\gamma_{P}S_{P}-\mu_{P}S_{P}$$

$$\frac{dI_{P}}{dt}=\gamma_{P}S_{P}-\mu_{P}I_{P}-\varepsilon I_{P}$$

$$\frac{dS_{PM}}{dt}=\varepsilon S_{P}-\mu_{M}S_{PM}$$

$$\frac{dI_{PM}}{dt}=\varepsilon I_{P}-\mu_{M}I_{PM}$$

Humans

$$\frac{dS_{H}}{dt}=b_{H}N_{H}+\omega I_{H1}+\theta I_{H2}-\gamma_{M}S_{H}-\gamma_{F}S_{H}-\mu_{H}S_{H}$$

$$\frac{dE_{H}}{dt}=\gamma_{M}S_{H}-\pi E_{H}-\mu_{H}E_{H}$$

$$\frac{dI_{H1}}{dt}=\pi E_{H}-\omega I_{H1}-\gamma_{A}I_{H1}-\mu_{H}I_{H1}$$

$$\frac{dI_{H2}}{dt}=\gamma_{F}S_{H}+\gamma_{A}I_{H1}-\theta I_{H2}-\mu_{H}I_{H2}$$

Environment

$$\frac{dE_{T}}{dt}=\psi\delta l(I_{H1}+\alpha I_{H2})-\rho E_{T}$$
