# Supplementary material for: Data-driven analyses of behavioral strategies to eliminate cysticercosis in sub-Saharan Africa
Source: PLoS Negl Trop Dis. 2021 Mar 23;15(3):e0009234. doi: 10.1371/journal.pntd.0009234 (PMC8018642; doi:10.1371/journal.pntd.0009234)
Supplement: S1 Text — A Description of Derivation of Slaughter Rate for Domestic Pigs (ε). B Dimensional Analysis for Birth Rate for Domestic Pigs (bP). (DOCX) [file pntd.0009234.s002.docx]

**S1 Text**

**S1 Text A** Description of Derivation of Slaughter Rate for Domestic Pigs (ε)

The death rate of pigs that survive to slaughter (representing approximately 73% of pigs alive at any point in time) is as follows: 60% are piglets and 40% are sows. Among the piglets, 90% are home-slaughtered at age 1 year and 10% are slaughtered at age 1.5 years. These estimates come from expert opinion and our analyses of pigs slaughtered at the Koudougou abattoir in Sanguié. We assume that the sows are slaughtered at age 4 years based on ref [29] and expert opinion (S. Dallaire). This results in an age expectancy of 2.23 years old for pigs surviving to slaughter, for a death rate of 0.45 (1/2.23) death per pig-year. Among all pigs alive at any point in time, 74% are pigs who will survive to slaughter. This is based on the following: among 7 piglets born per sow-year (based on local expertise (Ganaba), we assume that in the rural setting each sow of reproductive age farrows once per year and the number of piglets is based on ref [29]), 50% will die before weaning at age 0.17 years (based on ref [29] and our data), 20% will die at age 0.5 years between weaning and slaughter (death rate in this period is not available but assumed from the age distribution of pigs alive at the time of our surveys), and 30% will survive to slaughter (including sows kept for reproduction).

If 74% of pigs survive to slaughter then 26% will die before slaughter due to other reasons. Thus, overall, 12%, 14% and 74% of piglets dying before weaning, dying between weaning and slaughter and being slaughtered (including sows kept for reproduction).  Our derivation of this breakdown is further described in S1 Table.

**S1 Text B** Dimensional Analysis for Birth Rate for Domestic Pigs (b_P_)

**Definitions:**

b_P_: Birth rate for domestic pigs

sz: Annual litter size per farrowing

f: Annual number of farrowings per sow

P_sows_: Proportion of pig population that are sows

**Units:**

b_P_: new piglets per pig per year

sz: piglets per farrowing

f: farrowing per sow per year

P_sows_: sow per pig population

**Equation:**

$$b_{P}=sz \times f\times P_{sows}$$

**Dimensional Analysis:**

New piglets per pig per year = $\frac{piglets}{farrowing}\times\left[ \left( \frac{farrowing}{sow} \right)/year \right]\times\frac{sows}{pig population}$

New piglets per pig per year = $\left[ \left( \frac{piglets}{total pig population} \right)/year \right]$
