# Supplementary material for: Data-driven analyses of behavioral strategies to eliminate cysticercosis in sub-Saharan Africa
Source: PLoS Negl Trop Dis. 2021 Mar 23;15(3):e0009234. doi: 10.1371/journal.pntd.0009234 (PMC8018642; doi:10.1371/journal.pntd.0009234)
Supplement: S1 Table — Description of Derivation of Slaughter Rate for Domestic Pigs (ε). (DOCX) [file pntd.0009234.s003.docx]

**S1 Table. Derivation of breakdown for the pig population by survival time**

| **Pig population group** | **Pig population subgroup** | **% of pigs born** | **Average age at death or slaughter (years)** | **Weighted life expectancy in the population^1^** | **Percentage alive at any point^2^**$\ddagger$ |
| --- | --- | --- | --- | --- | --- |
| Piglets dying before weaning |  | 50% | 0.17 (FAO 2012^3^ and our data) | 0.08 | 11.8% |
| Piglets dying between weaning and slaughter age |  | 20% | 0.5 | 0.10 | 14.7% |
| Pigs surviving to slaughter | Pigs slaughtered in abattoir | 30% * 10% * 80%^3^ = 2.4% | 1.5 | 0.04 | 73.5% |
|  | Pigs slaughtered at home | 30% * 90% * 80%^4^ = 21.6% | 1 | 0.22 |  |
|  | Sows slaughtered | 30% * 20% = 6%^4^ | 4 (FAO 2012^3^) | 0.24 |  |

^1^Estimated by multiplying the age at slaughter or death by the percentage of pigs born.

^2^Calculated by dividing the weighted life expectancy in each subgroup by the sum of the weighted life expectancies across all groups

^2^ This assumes that of the 30% of pigs surviving to at least the age of slaughter, 80% are slaughtered for meat, and 90% are slaughtered at home while 10% are slaughtered in abattoirs.

^3^ Reference: Food and Agricultural Organisation (FAO). Secteur Porcin Burkina Faso. Revues Nationales de l’élevage de la division de la production et de la santé animale de la FAO. 2012. No. 1 Rome.

^4^ This assumes that of the 30% of pigs surviving to at least the age of slaughter, 20% are kept for reproduction

$\ddagger$ Rounded to sum to 100%
