# Supplementary material for: Data-driven analyses of behavioral strategies to eliminate cysticercosis in sub-Saharan Africa
Source: PLoS Negl Trop Dis. 2021 Mar 23;15(3):e0009234. doi: 10.1371/journal.pntd.0009234 (PMC8018642; doi:10.1371/journal.pntd.0009234)
Supplement: Attachment — Submitted filename: Response to Reviewers_PLOS NTDs Cysticercosis.docx [file pntd.0009234.s004.docx]

December 9, 2020

Dear Siddhartha Mahanty and Marco Coral-Almeida:

We wish to thank the reviewers for the insightful and helpful comments on our manuscript. We are thus pleased to submit a revised version of the paper, “Data-driven analyses of behavioral strategies to eliminate cysticercosis in sub-Saharan Africa,” as invited by the decision letter for PNTD-D-20-01164. We have modified the manuscript to address the comments and suggestions of the reviewers as detailed below. Please find each of the reviewers’ comments in *italics* and our responses in normal face.

Thank you for your consideration of our revised manuscript. We look forward to hearing from you.

Sincerely,

Hélène Carabin, DVM, MSc, PhD

Université de Montréal

*Reviewer #1:*

*1. The manuscript "Data-driven analyses of behavioral strategies to eliminate cysticercosis in sub-Saharan Africa" offers an interesting and innovative methodology to study disease dynamic interactions based on community data. The reported results offer sufficient evidence to implement feasible strategies at a taeniasis/cysticercosis control program for researchers and decision-makers. The data offered by authors probably is unique to estimate worthing information of epidemiological parameters.*

Thank you for the thoughtful feedback. We are eager to share the work and indeed hope that the results guide researchers and decision-makers.

*2. However, I found that a small set of model parameters probably need to be corrected and probably redefined. Specifically in pig demography. Birth rate according to the data each sow gives birth 7 piglets per year, which means that for two years a sow will have 14 piglets and she will be 3 years old (so a probable rate 14/3 almost 5 times 0.2 because of adults, but this rate easily can be twice or even be triple value in commercial farms).*

Thank you for this comment. It is true that these productivity estimates are very low, but it is the sad reality of small-scale farmers in very poor settings such as Burkina Faso. We based our estimates on evidence we could find from surveys conducted for FAO and data from pig farmers in our own study. Therefore, we are quite confident that these values are the best estimates we can get for this setting. Sows do not have access to quality food; very few boars are available for reproduction (it is not uncommon to see one boar for a whole village); and there is no program to improve the genetics of the animals, all which also explains in part this poor productivity estimate. More details on the reproduction of pigs by smallholding farmers in Burkina Faso are available in Ref. 29, FAO of the manuscript.

*3. Likewise, a dimensional analysis will be needed in the parameter definition.*

Thank you for suggesting a dimensional analysis for the birth rate parameter. We have incorporated this in the Supplementary Material.

*4. Additionally, the sampling strategy used would be better explained in a figure.*

We have provided the reader with other references where the sampling strategy was explained. Because this manuscript uses data from three components of the parent study, it could become cumbersome to present a figure for the sample strategy and detract from the focus on the model here. We therefore do not feel it is necessary to repeat the description of the sampling strategy in this manuscript, as it has been explained in the other manuscripts (see Ref 22).

*5. It is necessary to mention that the model is a set of differential equations.*

We appreciate the recommendation and have added the following description to the Dynamic Transmission Model subsection of the Methods: “A deterministic, compartmental model for transmission in the multi-host, cysticercosis-taeniosis system was developed as a set of differential equations, with separate compartment structures for pigs and humans.”

*6. Running time should be mentioned to reach endemic conditions or at least mention the time when reported results were obtained.*

We appreciate the suggestion and have added the following clarification to the Model fitting algorithm section of the Methods: “For all iterations of model implementation, the model was run for 10,000 timesteps (*i.e*., months) and the results were evaluated to assess whether endemic conditions had been reached. If the difference in prevalence of cysticercosis (I_H2_) in the human population at t=10,000 and t=9,990 was <0.000001, the model was assumed to have reached dynamic equilibrium. If the condition was not met, the model was run for an additional 10,000 timesteps and re-evaluated. The process was repeated iteratively for a maximum 50,000 timesteps, and all iterations converged to equilibrium within that period.”

*7. L44 and elsewhere, citations and references are after final sentence dot.*

Thank you. We have reformatted the submission to reflect the reference guidelines of *PLOS NTDs*.

*8. L201: mention that the model is a set of differential equations*

Thank you again for the suggestion. The sentence has been modified as indicated in response to comment #5 above.

*9. L335 is Eq 7?*

You are correct. We have updated the text and now reference Eq 7 when describing the likelihood equation.

*10. Is it possible to mention which pork-made dishes are prepared and why they could contain undercooked pork?*

Thank you for suggesting more context around pork dishes in Burkina Faso. In the Methods section, we have detailed the types of pork dishes and modes of preparation common in our study setting: “Market-based pork meals in Burkina Faso include consumption of roasted meat (*porc-au-four*) or meat stews from street vendors and cookshops. Meat is often prepared in mud ovens for varying periods of time and at varying levels of heat.”

*Reviewer #2:*

*11. This article presents a deterministic multi-host (humans and pigs) compartmental model for the transmission of different stages of the tapeworm Taenia solium in a closed population. The purpose of the study presented is to consider the impact of less resource intensive interventions focused on human behaviours to eliminate transmission as a public health problem. The authors point out the importance of utilising large field-based study data to inform such interventions. Overall, I think this study is of public health relevance and is a good starting point to addressing the gaps in currently available models of taeniosis/cysticercosis and the impacts of interventions on elimination of the disease as a public health problem. I have detailed comments below which need to be addressed prior to the publication of this manuscript.*

We thank the reviewer for recognizing the potential of this study and future work that builds upon it.

*12. The authors refer to elimination and define it as less than one case per 1,000 population. My understanding would be that this refers to “elimination as a public health problem”, if this is the case the authors should clarify this within their article.*

Thank you for highlighting the need for clarification. We now state in the Methods section that, “For each intervention approach, the reduction in transmission associated to below a threshold of one case per 1,000 population was determined. In the absence of an elimination target from the World Health Organization, we defined this threshold for ‘elimination as a public health problem’.”

*13. The article is lacking a clear and concise description of what the authors refer to as “taeniosis/cysticercosis” (note that in the author summary only ‘cysticercosis’ is referred to). This needs to be incorporated more clearly into the introduction to give readers a clear idea of the disease in question . E.g. incorporate a description of the parasite lifecycle and the link between taenoiosis and cysticercosis – this could be written description or inclusion of a figure such as the one here:* [*https://www.cdc.gov/parasites/cysticercosis/biology.html*](https://www.cdc.gov/parasites/cysticercosis/biology.html)

*Some of this information is presented under the dynamic transmission model section, but it needs to be explained earlier in the article than this to ensure you don’t lose readers, for example by referring to parasite stages (eg proglottids) before explaining why those stages are important.*

We appreciate the recommendation. The following description, along with reference to the CDC website, has been added to the first paragraph of the Introduction: “Human cysticercosis is a condition that results as cysticerci, or larvae of the parasite *Taenia solium* (*T. solium*), develop in the body’s tissues after people ingest *T. solium* eggs, and thus act as accidental intermediate hosts [2]. The usual life cycle of the parasite however involves pigs as intermediate hosts, and humans as final hosts. Pigs can develop cysticercosis upon ingestion of *T. solium* eggs, facilitated by free-roaming pig production practices and poor sanitation. Consumption of undercooked pork dishes prepared from infected pigs, in turn leads to *T. solium* taeniosis in humans, i.e. infection with the adult form of the tapeworm. Tapeworm carriers excrete parasite eggs when gravid proglottids are passed with the stool. Exposure to *T. solium* eggs, either in the environment, or through direct contact with taeniosis-infected individuals or self-infection, facilitated by open defecation, poor access to sanitation and hygiene, can lead to human cysticercosis.”

*14. General comment: at first mention of Taenia solium put in brackets (T. solium) before using abbreviation throughout. Do not assume the reader will know what the abbreviation stands for.*

Thank you for mentioning this oversight. We have added ‘*(T. solium)’* after the first mention of *Taenia solium* in the Introduction (line 4 of the section).

*15. Question from assumption on line 227: do the authors think it is it realistic to assume that susceptible and infected pigs were slaughtered at equal rates, or are infected pigs likely to show signs of illness and be culled earlier or removed from production?*

Thank you for this comment. Many pigs infected with cysticercosis will show very little signs of disease. Moreover, traditional pig farming with pigs left roaming to scavenge for their food constitute 90% of all pig raising in the country, a proportion nearly 100% in the studied villages. In this context, even if pigs would exhibit signs, they may not be noticed by the farmers. Furthermore, there is no national plan for training pig farmers, traders, butchers, foodshop owners on biosecurity of diseases affecting pigs. Meat inspection and public veterinary services are lacking in the country with estimated 92% and 97% of pigs are home slaughtered without any inspection in Boulkiemdé and Sanguié, respectively (see ref 29, FAO, in the manuscript for more details on pig production in Burkina Faso). Moreover, pigs are often slaughtered once an elder dies or for other special occasions. They may also be slaughtered when the family needs money or the pigs can no longer find food. So the decision to slaughter an animal is not directly linked to its growth or size, but more to an immediate need. Therefore, we feel that there is no strong link between cysticercosis and the probability of slaughter.

*16. Thorough description of the transmission model. The authors provide a transparent description of assumptions made about model parameters and the way they have incorporated the impact of interventions on reducing transmission.*

We appreciate the recognition of our efforts to transparently communicate all model assumptions.

*17. Line 332: Comment on the limitation or impact the use of aggregate quantities may have on derived estimates and model outcomes?*

We agree with the reviewer that the use of aggregate quantities may impact the findings that can be derived from the study. We now state in the Limitations section of the Discussion: “The lack of data to inform individual parameters led to decisions around the structure and interpretation of our model. Specifically, the use of aggregate parameters was intended to maintain the model structure with all individual parameter components defined so that it can be used to answer more nuanced questions in the future, as additional data to inform components become available. However, by fitting aggregate quantities, interpretation of the parameter distributions—particularly the absolute values—is limited and it is difficult to pinpoint exact relationships across provinces for the individual component parameters.”

*18. Line 335: typo, I assume the likelihood equation is actually Equation (7) on line 320?*

Thank you for identifying the typo. We have corrected the equation number for the reference to the likelihood equation.

*19. Line 356 should refer to average prevalence of active human cysticercosis to align with Figure 2(a) label.*

We thank the review for catching this oversight. The first sentence in the Results section ‘Model fit and province-specific parameterization’ has been rewritten as follows: “On average, the model-predicted average prevalence of active human cysticercosis and current human taeniosis for all provinces and across parameter sets was reflective of the data, in terms of both point estimates and confidence intervals (Figure 2).”

*20. Figure 2 is a nice illustration/comparison of modelled outputs compared to data. Consider changing the y-axis to percentages for readability (e.g. 0%, 5%, 10%, 15% instead of 0, 0.1, 0.15).*

Thank you for the suggestion. We have changed the y-axis of Figure 2 to percentages.

*21. Figure 3 needs a legend to indicate which province is represented by blue, yellow and green estimates.*

A legend depicting the color coding by province has been added to Figure 3.

*22. Figure 5 caption does NOT align with the figure. E.g. looking at Figure 5A and the labels therein I would interpret this as: 5% reduction in exposure to T. solium cysticerci in combination with a 5 – 50% reduction in exposure to T. solium eggs. This is NOT what is written in the Figure 5 caption on lines 443 – 452. The same comment goes for Figures 5B, 5C and 5D.*

*From the discussion text I think that it is the figure 5 caption which is incorrect.*

Thank you for the comment. We have corrected the percentages for the Figure 5 legend as follows: “Figure 5. Combined impact of pork cooking and latrine use interventions on active cysticercosis. (A) 5% reduction in exposure to *T. solium* cysticerci in undercooked pork meat, in combination with 5-50% reductions in exposure to *T. solium* eggs in the environment. (B) 10% reduction in exposure to *T. solium* cysticerci in undercooked pork meat, in combination with 5-50% reductions in exposure to *T. solium* eggs in the environment. (C) 25% reduction in exposure to *T. solium* cysticerci in undercooked pork meat, in combination with 5-50% reductions in exposure to *T. solium* eggs in the environment. (D) 50% reduction in exposure to *T. solium* cysticerci in undercooked pork meat, in combination with 5-50% reductions in exposure to *T. solium* eggs in the environment. “

*23. How was the elimination threshold of less than one case per 1,000 population decided upon?*

In the absence of an elimination target for cysticercosis from the World Health Organization, we chose a target consistent with that proposed for elimination as a public health problem of other neglected tropical diseases, such as trachoma.

*Reviewer #3:*

*24. This is an important paper that, as identified by the authors, contributes to filling an gap in the area of T. solium research (developing transmission models informed by field-data). The model also provides a novel contribution by robustly assessing the impact of non-pharmaceutical interventions, with most T. solium models to date focusing on pharmaceutical interventions. In terms of contribution to understanding prospects for control, the paper considers interventions which might be more feasible/sustainable in severely resourced endemic settings.*

*The approach adopted uses data from a detailed study in Burkina Faso (with pre-randomisation baseline prevalence data, adjusted for diagnostic performance in a latent class model). A Bayesian Melding model fitting approach samples key parameters including the rate of loss of viable eggs (which is an important parameter to elucidate as very minimal data is available), two transmission coefficients for human taeniosis and porcine cysticercosis, and the ratio of human contact rate with eggs with pig contact rate with eggs. After fitting, the contact rates are identified as higher in Nayala compared to the other two provinces, while the rate of loss of viable eggs is similar across settings. Reduction in exposure to T. solium eggs is presented as a more effective intervention, compared to reducing consumption of undercooked meat at reaching human active cysticercosis elimination thresholds.*

*The differences in modelled contact rates between provinces eloquently explain the different effectiveness of modelled interventions between provinces, supporting the observations of heterogeneous intervention effectiveness in the field trial conducted in Burkina Faso by the same group.*

*Overall, I very much like this manuscript and think it will be an important contribution. I do however has some important points to raise below.*

We appreciate the reviewer’s comments and have addressed the points raised below.

*25. Including data/text on burden of disease in the introduction paragraph 2 (lines 46-50) to help contextualize the clinical aspect for more general audiences.*

We have added the estimate obtained by the FERG and published in 2015 in the Introduction paragraph 2: “*T. solium* has been estimated to incur 2.78 million DALYS in 2010, ranking number 4 out of 33 foodborne diseases, and having the higher rate of DALYs per 100,000 population in sub-saharan Africa of all foodborne infections evaluated [6].”

*26. Table 1 - If I am understanding the methodology correctly, the prevalence estimates provided in Table 1 as the adjusted prevalence estimates obtained from the Latent class model, which are then used for model fitting. If this is the case, highlighting in Table 1 that these are adjusted prevalence estimates (not unadjusted), would improve clarity.*

Thank you for the suggestion. We have added a footnote to the table to indicate that the values for Prevalence of current taeniosis in humans and Prevalence of active cysticercosis/current taeniosis coinfection in humans are estimates from “Latent Class models, adjusting for misclassification error (ME)”.

*27. Table 2 - It is not clear to me how the slaughter rate for domestic pigs is calculated from the detail provided (and how the reference [24] Chembensofu et al. 2017 is used to inform this). Please could the authors clarify.*

These estimates were based on data collected from questionnaires to pig owners, ref [29] and expert opinion. We agree that we did not explain that clearly enough in the table and have added the following clarity to the Supplementary Material. The death rate of pigs that survive to slaughter (representing approximately 73% of pigs alive at any point in time) is as follows: 60% are piglets and 40% are sows. Among the piglets, 90% are home-slaughtered at age 1 year and 10% are slaughtered at age 1.5 years. These estimates come from expert opinion and our analyses of pigs slaughtered at the Koudougou abattoir in Sanguié. We assume that the sows are slaughtered at age 4 years based on ref [29] and expert opinion (S. Dallaire). This results in an age expectancy of 2.23 years old for pigs surviving to slaughter, for a death rate of 0.45 (1/2.23) death per pig-year. Among all pigs alive at any point in time, 74% are pigs who will survive to slaughter. This is based on the following: among 7 piglets born per sow-year (based on local expertise (Ganaba), we assume that in the rural setting each sow of reproductive age farrows once per year and the number of piglets is based on ref [29]), 50% will die before weaning at age 0.17 years (based on ref [29] and our data), 20% will die at age 0.5 years between weaning and slaughter (death rate in this period is not available but assumed from the age distribution of pigs alive at the time of our surveys), and 30% will survive to slaughter (including sows kept for reproduction) corresponding to 12%, 14% and 74% of piglets dying before weaning, dying between weaning and slaughter and being slaughtered (including sows kept for reproduction).

*28. Could the authors explain why a pre-patent compartment in pigs is omitted, where this duration (2- 3 months as demonstrated by Yoshino, 1933a,b) is non-negligble compared to the relatively short life-span of pigs in endemic settings :*

*Yoshino K. Studies on the post-embryonal development of Taenia solium, Pt. i. J Med Assoc Formosa 1933a;32:139–41.*

*Yoshino K. Studies on the Postembryonal Development of Taenia solium. Part II. On the Youngest Form of Cysticercus cellulosae and on the Migratory Course of the Oncosphaera of Taenia solium within the Intermediate Host. J Med Assoc Formosa 1933b;32:1569-1586.*

Our data from pig cysticercosis in this area suggest that infection occurs early in the life of a pig in this setting and is ongoing. Indeed, the prevalence was above 40% and did not increase after the age of 3 months. Therefore, even though there is a pre-patent period, most pigs that will become infected will have active cysts by age 1, 1.5 or 4 years old when they are slaughtered and consumed by humans. Therefore, ignoring the pre-patent period here will not have much of an impact on our results. We have added a sentence in the methods section to justify this.

*29. Could the authors explain their rationale behind not including heterogeneity in burden of porcine cysticercosis infection (e.g. low or high burden) which could influence the probability of infection for human taeniosis infection?*

The lack of data on infection burden in pigs, as well as the relative transmission probabilities influenced our decision to not include multiple compartments or distributional assumptions to account for the proportions of pigs with low versus high burden. We now state this in the Discussion as a limitation and indicate possible future steps: “Likewise, the current model does not account for heterogeneity in the burden of porcine cysticercosis infection. Without data on porcine parasite load or exposure to *T. solium* eggs (*i.e*., rate of ingestion as the distribution of dosage across pig populations in each province), future work could introduce distributional assumptions, starting with consideration of an overdispersed distribution to reflect differential probability of exposure to high versus low dosages or differential burden of parasite load across the porcine population. The sensitivity of the model results around human prevalence to distributional assumptions around differential burden or exposure among pigs could be explored.”

*30. The force-of-infection equation for human taeniosis (equation 2) indicates that human contact rate with pork is assumed to be frequency-dependent. It would be useful to explicitly state this, and similarly for human or pig contact rates with contaminated soil, where equations 1 and 4 indicate density-dependent contact rates are assumed.*

Thank you for the suggestion. In the revised manuscript, we have qualified the description of each force-of -infection equation with the terms “frequency-dependent” (for equation 2) or “density-dependent” (for equations 1 and 4).

*31. Could the authors clarify the assumption that pork consumed at home “ was always cooked at a temperature sufficient for killing parasites” on lines 244-245 compared to pork cooked in the market. Did they see this in their data/ study area?*

The authors did receive feedback consistent with this assumption in the study area. In particular, they learned that, while pork prepared at home is in the form of stews and boiled for long periods of time, that is not the case of pork served in cookshops and by street vendors. Our data from interviews of mothers show that 98.5% of women preparing pork for the household boils the meat (3107/3156 mothers preparing pork). As for pork sold in markets, given its popularity, pork meat is increasingly less cooked over the course of the day. We have noted this directly in the manuscript where the assumption is stated.

*32. I am not clear on the definition of seroreversion rate parameter (θ) on lines 280-281, and Table 2 - is this seroreversion from both human cysticercosis and human taeniasis seropositivity back to full susceptibility? Lines 280-281 makes me think this is only seroreversion from human cysticercosis? Please could the authors clarify.*

In our transmission model, seroreversion is being used to refer to the loss of cysticercosis infection in humans (either cysticercosis infection alone or co-infection with taeniosis). However, as our model structure reflects and as has been suggested by a subsequent comment, the parameter θ represents a transition from active cysticercosis infection to susceptibility, or natural loss of infection, regardless of loss of seropositivity. Accordingly, in the Methods, we now clarify: “Degeneration of all tissue cysts results in the movement of individuals to the susceptible compartment (S_H_) at a rate θ, which we define as loss of infection.” Additionally, within Table 2, parameter θ is now more specifically defined as, “Recovery after loss of cysticercosis infection.”

*33. Also, should θ be defined as the human recovery or infection loss rate (from true infection i.e IH2 to SH), as presumably the seroprevalence data is adjusted based on the diagnostic performance, so seroreversion can be interpreted as those who are truly reverting from infected status to susceptibility? I find the name of the parameter (the seroreversion rate) slightly confusing as the model captures true infection dynamics (not seropositivity)?*

We appreciate the clarifying question. As indicated in response to the previous comment, we have removed reference to seropositivity and seroreversion and redefined parameter θ in terms of loss of cysticercosis infection among humans.

*34. How are the interventions applied? Are they implemented as an instantaneous reduction in the rate of transmission by the factors mentioned on lines 301 and 308 and maintained throughout the duration of the intervention programme? Also, when is the modelled prevalence measured to assess the impact of the intervention – is this in line with the 18-month follow-up (post randomisation survey) indicated in ref [22]? This is not clear in the “Intervention approaches” section of the methods. There is a little text on modelling “pulse interventions” in the model limitations section (lines 502-503), however more information should be provided in the methods section.*

The following detail has been added to the ‘Intervention approaches’ section of the Methods: “For baseline (no intervention), the model was run to equilibrium using, as inputs, fixed parameters (Table 2) and acceptable posterior parameter sets identified during the fitting process and, as the output, the prevalence of active cysticercosis (per 1,000 population) at the equilibrium. For each intervention approach, the model was run with percent reductions in individual or multiple rates of transmission, according to our assumptions for how interventions would impact transmission, to represent the individual and combined intervention strategies, respectively, under consideration. The output of the intervention scenarios was likewise the prevalence of active human cysticercosis once the model reached equilibrium. In this way, we considered the long-term impact of routine and uniform implementation of interventions in endemic areas.”

*35. There appears to be an error in equation # 4 in the SI equation: should the addition term to dIpm/dt be εIP not εIPm?*

That is correct. We have fixed this error in the first term of equation #4.

*36. It is not clear what the subscript j refers to in model fitting algorithm section (lines 317 – 325), presumably where the model was fitted to each province, j represents each province? If so, could this be explicitly written (i.e. “For each of the provinces (j), four..”*

We have incorporated this suggestion and updated the statement to read, “For each of the three provinces (j), four quantities…”

*37. Could the authors provide justification for the assumption that the contact rate of pigs with eggs in the environment (χP) is always larger than the contact rate of humans with eggs in the environment/ with other taeniosis carriers (χF) or autoinfection (χA), given the parameter estimates outlined in line 340 for model fitting and the information outlined in Table 2?*

We have included justification as a footnote to Table 2 where the assumption is indicated: “This assumption was based on the authors’ understanding of behavioral exposure to soil contaminated with *T. solium* eggs for pigs versus humans. Since pigs spend the majority of time roaming to find their own food (in the dry season) or tethered (in the short wet season) with regular exposure to sources of environmental contamination, they were expected to have higher contact with *T. solium* eggs than humans, whose environmental exposure would be, for example, from latrine use, shaking contaminated hands with an infected individual, or consumption of contaminated food—behaviors being intermittent throughout the day and at dosage levels less than walking or laying on contaminated soil.”

*37. I find the Figure 5 legend slightly confusing – is 5A not a 5% reduction in consumption rather than 20% in the legend text on line 444 for example?*

The reviewer is correct. We have rewritten the Figure 5 legend to reflect the percentages indicated in the figure itself.

*38. The authors mention the added value of this paper in presenting “field-driven force-of-infection” modelling. It would be useful if the authors could present in the manuscript the specific province-level FoI estimates calculated in the model at endemic equilibrium.*

Thank you for the suggestion. We have opted not to include the FoI estimates since they were not considered to be a major outcome of the model and may not be generalizable to other models unless they are structured in the same way as our model. A detailed description of their derivation, which we provide in our methods, was perceived to be a more important contribution for future reproducibility or adaptation. Our mention of the use of ‘field-driven force-of-infection’ modelling was intended to emphasize the added value of dynamic modelling (versus statistical modeling) to be able to account for changes in transmission due to intervention and represent outcomes such as reduction in incidence to below a prescribed threshold. Importantly, the use of field data and experience to inform the model enhances the relevance of the research question and model structure to feasible, context-specific research questions.

*39. Do the authors have any idea why the modelled contact rates are higher in Nayala, while the pre-intervention prevalence is the highest in Boulkiemdé? Would it not expected that a higher baseline T. solium prevalence would be found in a setting with higher contact rates (Nayala)? The authors begin to elude to this in lines 477-480, but I think more discussion should be provided.*

We thank the reviewer for the comment. Based on the findings, we noted that Nayala had a higher average value for the aggregate parameter representing the product of the probability of transmission of *T. solium* cysticerci from pork meat to humans and the rate of undercooking in markets ($\beta_{M}c_{k}$). It was thus inferred that the rate of undercooking in Nayala was higher than that in Boulkiemdé. We have added to the Discussion that, “Since Nayala had a lower baseline prevalence of active cysticercosis in pigs than in Boulkiemdé, a higher rate of undercooking did not necessarily translate into a higher prevalence of taenioisis or human cysticercosis in Nayala.” Based on the observations, we further note that “Pig-focused interventions could have greater impact in Boulkiemdé.”

*40. The rate of loss of viable eggs in the environment is an important parameter, where minimal data is available from field settings (most models relying on data from other Taenia species). In Figure 3, the authors find a similar rate of loss of viable eggs in environment in each province. Is this expected? perhaps the authors could discuss more about the similarity (or not) of environmental conditions across provinces which could lead to these findings.*

We agree that the rate of loss of viability is important. The similarity in the parameter distribution across the three provinces was not unexpected. Monthly rate of decay of egg viability is expected to be largely related to climate and biology and partly related to egg removal such as through baseline sanitation and hygiene practices. The three provinces in the study area do not vary significantly in terms of climate, and environmental hygiene and sanitation practices were not observed to differ in a notable way. However, this is something that would be extremely interesting to study if valid and affordable tools to measure eggs in the environment were to become readily available.

*41. In results, it is stated that "βE is a biological parameter that could be assumed to uniform across provinces", however the probability of viable infection upon contact (with infective material) may vary on the basis of whether pigs are exposed to smaller doses e.g. contacting eggs in the soil (lower probability) compared to higher doses e.g. consuming proglottids (higher probability). Santamaria et al. 2002 (ref below) indicate that the efficiency of establishment (proportion of viable cyst developing) is influenced by the dose (eggs consumed). Have the authors considered this?*

Thank you for the reference. As mentioned in response to a previous reviewer comment, future work could incorporate assumptions around differential exposure, which could then be linked to differential infection intensity and transmission probability. We have noted this in the Limitations section of the Discussion.

*42. Table 1: It is not clear what ME is, could the authors please define this.*

A clarifying footnote has been added to Table 1 to clarify that rows 2 and 3 reflect “Estimates from Latent Class models, adjusting for misclassification error (ME).”

*43. Table 1: What does Sp represent in the footing of Table 1 (line 133) – I think it refers to susceptible pigs as as per the model figure, but this has not been defined yet in the text.*

Thank you for identifying the lack of clarity. Sp means specificity here. We have clarified

the matter in Table 1.

*44. Table 2: It would be easier for the reader if another column was made available in Table 2 to indicate the standardized monthly rate for parameter estimates.*

We agree with the reviewer and have added the column of standardized monthly rates for parameter estimates.

*45. In Figure 3, it would be useful to include a legend for colour of the points based on each province for quick reference (otherwise there is a need to constantly refer back to Fig 2 to check)*

The point is well taken. A legend has been added to Figure 3.

*46. Line 335: this should refer to equation 7 for the likelihood equation*

That is correct. Thank you for identifying the typo. It has been corrected in the revised manuscript.
