# Supplementary material for: Data-driven analyses of behavioral strategies to eliminate cysticercosis in sub-Saharan Africa
Source: PLoS Negl Trop Dis. 2021 Mar 23;15(3):e0009234. doi: 10.1371/journal.pntd.0009234 (PMC8018642; doi:10.1371/journal.pntd.0009234)
Supplement: Attachment — Submitted filename: Cysticercosis_PLOS NTDs_Response to Reviewers v2_FNL.docx [file pntd.0009234.s005.docx]

January 28, 2020

Dear Siddhartha Mahanty and Marco Coral-Almeida:

We once again wish to thank the reviewers for the time to consider our revised manuscript and the additional feedback. We are thus pleased to submit a further revised version of the paper, “Data-driven analyses of behavioral strategies to eliminate cysticercosis in sub-Saharan Africa,” as invited by the decision letter for PNTD-D-20-01164R1. The manuscript to address the comments and suggestions of the reviewers as detailed below. Please find each of the reviewers’ comments in *italics* and our responses in normal face.

Thank you for your consideration of our revised manuscript. We look forward to hearing from you.

Sincerely,

Hélène Carabin, DVM, MSc, PhD

Université de Montréal

*Reviewer #1:*

Authors properly have managed the questions related to the estimation of epidemiological parameters and therefore, I think, the model may be a useful tool with proper parameter values necessary to understand this disease dynamics and to manage strategies for disease control and elimination in the communities. Therefore I recommend accepting it to publish in PLOS NTD. I have suggested just to include the software used for the model running.

We appreciate the positive feedback on the revised manuscript. Referencing the

software [Matlab version R2016a (9.0.0)] used for the implementation of the

transmission model is a helpful suggestion, which has been incorporated into the first

paragraph of the Methods section.

Reviewer #2:

By revising this manuscript and addressing concerns highlighted by the reviewers in detail, the authors have produced an interesting manuscript of public health relevance which is now fit for publication.

Thank you. We are eager to have the paper and results shared with the broader public

health community to maximize the potential for impact of the tool in terms of being

applied to inform other researchers, practitioners, and decision makers.

Reviewer #3:

Firstly, I would like to thank the authors on their detailed responses, and congratulate the authors on an improved manuscript.

We are grateful for the reviewer’s consideration of the revised manuscript and continued

recommendations for improvement.

Before proceeding, I would like to first request clarification on the following responses please:

In response to point 27:

Thank you for further clarification on the slaughter rate, this is much clearer now. Could the authors walk me through their derivation of 12;14;74% dying for the three different reasons mentioned in the supplementary paragraph below:

"Among all pigs alive at any point in time, 74% are pigs who will survive to slaughter. This is based on the following: among 7 piglets born per sow-year (based on local expertise (Ganaba), we assume that in the rural setting each sow of reproductive age farrows once per year and the number of piglets is based on ref [29]), 50% will die before weaning at age 0.17 years (based on ref [29] and our data), 20% will die at age 0.5 years between weaning and slaughter (death rate in this period is not available but assumed from the age distribution of pigs alive at the time of our surveys), and 30% will survive to slaughter (including sows kept for reproduction) corresponding to 12%, 14% and 74% of piglets dying before weaning, dying between weaning and slaughter and being slaughtered (including sows kept for reproduction)."

We have added a Table in the Supplementary Material (Section S2.2) to explain how these estimates were obtained. We hope that this will clarify the approach.

**Table. Derivation of breakdown for the pig population by survival time**

| **Pig population group** | **Pig population subgroup** | **% of pigs born** | **Average age at death or slaughter (years)** | **Weighted life expectancy in the population^1^** | **Percentage alive at any point^2^**$\ddagger$ |
| --- | --- | --- | --- | --- | --- |
| Piglets dying before weaning |  | 50% | 0.17 ([29] and our data) | 0.08 | 11.8% |
| Piglets dying between weaning and slaughter age |  | 20% | 0.5 | 0.10 | 14.7% |
| Pigs surviving to slaughter | Pigs slaughtered in abattoir | 30% * 10% * 80%^3^ = 2.4% | 1.5 | 0.04 | 73.5% |
|  | Pigs slaughtered at home | 30% * 90% * 80%^3^ = 21.6% | 1 | 0.22 |  |
|  | Sows slaughtered | 30% * 20% = 6%^4^ | 4 [29] | 0.24 |  |

^1^Estimated by multiplying the age at slaughter or death by the percentage of pigs born.

^2^Calculated by dividing the weighted life expectancy in each subgroup by the sum of the weighted life expectancies across all groups

^2^ This assumes that of the 30% of pigs surviving to at least the age of slaughter, 80% are slaughtered for meat, and 90% are slaughtered at home while 10% are slaughtered in abattoirs.

^3^ This assumes that of the 30% of pigs surviving to at least the age of slaughter, 20% are kept for reproduction

$\ddagger$ Rounded to sum to 100%

In response to point 28:

If I am understanding correctly, does this not indicate that non-infected pigs largely become resistant after age of 3 months (with continued exposure), if (age-?)prevalence does not increase from 40% after 3-months of age (assuming constant exposure with age)? As this is not an age-structured model or does not include parameters for immunity, the authors will not be able to plot an age-prevalence curve to see if the model captures these age-infection dynamics. However, the model presented without age-structuring here presumably models a constant force-of-infection with respect to age, therefore the prevalence will increase with age. I want to authors to comment on this, given they mention in the manuscript the prevalence doesn’t rise from 3 months of age and their model will not presumably capture their own field observations (of prevalence saturating with age; therefore the current model assumes prevalence keeps increasing with age, so slaughter age-animals will have a higher prevalence than those at 3-months of age), and have not included parameters for immunity (which may lead to an underestimation in the force-of-infection). See Lewis and Torgerson 2014 (Echinoccocus multilocularis), and Torgerson et al 1998 (Taenia hydatigena) which use a simpler catalytic model formulation fitted to age-prevalence curves for further reference.

- Lewis, F.I., Otero-Abad, B., Hegglin, D., Deplazes, P. and Torgerson, P.R., 2014. Dynamics of the force of infection: insights from Echinococcus multilocularis infection in foxes. PLoS neglected tropical diseases, 8(3) e2731.

- Torgerson, P.R., Williams, D.H. and Abo-Shehada, M.N., 1998. Modelling the prevalence of Echinococcus and Taenia species in small ruminants of different ages in northern Jordan. Veterinary Parasitology, 79(1), pp.35-51.

You are correct that the force of infection does not reflect age-specific contributions to transmission by infected pigs. The current model, since implemented at the population level without age stratification, does not account for differential infection and therefore infectiousness by age. Instead, the transmission rate represents the average rate of transmission within the entire pig population. Likewise, the susceptibility is not changing, such that all pigs, on average, are susceptible to infection at a given rate. We have noted in the Discussion section that ‘The force of infection in the pig population does not reflect age-specific contributions to transmission by infected pigs. Future iterations of the model, given available data, might be structured to capture age dynamics of transmission in pig hosts and not overestimate the contribution to transmission of slaughter-age pigs. While the added complexity may not be relevant in all cases, it could have implications for the evaluation of pig-targeted interventions.’

In response to points 32 & 33:

I still find this this a little unclear- as the authors response suggest, can they authors remove the “from seropositivity” and “(seroreversion) in the “Recovery from seropositivity after loss of cysticercosis infection (seroreversion)” for parameter θ in table 2, and similarly on line 313 of the revised manuscript, removing “seroreversion”.

We have addressed the reviewer’s concern. The parameter description for θ in Table 2 has been revised as requested and now reads: “Recovery after loss of cysticercosis infection.” Likewise, line 313 of the manuscript includes a reference to θ as “Degeneration of all tissue cysts results in the movement of individuals to the susceptible compartment ($S_{H}$) at a rate θ, which we define as loss of infection,” which reflects removal of the word “seroreversion.”

In response to point 34:

With the intervention simulations run to equilibrium, can the authors state the timeframe(s) to reach equilibrium under the different intervention strategies (is there significant variation for example if I am understanding correctly)? Are these within a realistic timeframe (i.e. if the simulations have taken a significant time to reach equilibrium, is it realistic that interventions/behaviour changes would be maintained for so long)?

We thank the reviewer for following up on this edit. In dynamic models defined by a system of differential equations, dynamic equilibrium reflects stability of solutions of the differential equations; therefore, whether a model reaches equilibrium and how long it takes to achieve it have more consequences computationally than in the context of the research question.

Our modeling approach was not designed to account for how long it would take for interventions to reduce prevalence to below the elimination threshold. It was being used to consider whether transmission in a setting with the intervention practices and defined behavior levels would reduce prevalence (at its dynamic endemic state) to below the threshold. If we had intended to evaluate the former as well, we would have run the model using the baseline parameters to equilibrium and then updated the parameters to represent introduction of the intervention into the baseline endemic state.

Recognizing the potential for misunderstanding, we have aimed to clarify this in the last sentence of the “Intervention approaches” section of the Methods: “The output of the intervention scenarios was likewise the prevalence of active human cysticercosis once the model reached equilibrium. In this way, we considered the long-term impact of routine and uniform implementation of interventions in endemic areas rather than the impact of pulse implementation or of time-dependent changes in implementation coverage.”
